# Monolayers of MoS_2_ on Ag(111) as decoupling layers for organic molecules: resolution of electronic and vibronic states of TCNQ

**DOI:** 10.3762/bjnano.11.91

**Published:** 2020-07-20

**Authors:** Asieh Yousofnejad, Gaël Reecht, Nils Krane, Christian Lotze, Katharina J Franke

**Affiliations:** 1 Fachbereich Physik, Freie Universität Berlin, Arnimallee 14, 14195 Berlin, Germany

**Keywords:** decoupling layer, molybdenum disulfide (MoS_2_), scanning tunneling microscopy, tetracyanoquinodimethane (TCNQ), vibronic states

## Abstract

The electronic structure of molecules on metal surfaces is largely determined by hybridization and screening by the substrate electrons. As a result, the energy levels are significantly broadened and molecular properties, such as vibrations are hidden within the spectral line shapes. Insertion of thin decoupling layers reduces the line widths and may give access to the resolution of electronic and vibronic states of an almost isolated molecule. Here, we use scanning tunneling microscopy and spectroscopy to show that a single layer of MoS_2_ on Ag(111) exhibits a semiconducting bandgap, which may prevent molecular states from strong interactions with the metal substrate. We show that the lowest unoccupied molecular orbital (LUMO) of tetracyanoquinodimethane (TCNQ) molecules is significantly narrower than on the bare substrate and that it is accompanied by a characteristic satellite structure. Employing simple calculations within the Franck–Condon model, we reveal their vibronic origin and identify the modes with strong electron–phonon coupling.

## Introduction

When molecules are adsorbed on metal surfaces, their electronic states are strongly perturbed by hybridization, charge transfer and screening [1–4]. These effects lead to a broadening and shift of the molecular resonances [5]. Often the molecular functionality is also lost due to these interactions [6]. However, addressing individual molecules in devices or by single-molecule spectroscopy as offered in a scanning tunneling microscope, requires a metal electrode. To (partially) preserve the molecular properties the molecule–electrode coupling has to be properly designed. An elegant way is to clamp the molecule between electrodes via single-atom bonds at opposing sites of the molecule while the molecule is freely hanging between the electrodes [7–10]. While these configurations give access to important transport properties [11–13], they do not allow for imaging molecular properties with intramolecular resolution [14]. The latter requires the molecules to be flat lying on a surface. To decouple such flat-lying molecules from a metal, thin insulating layers have been engineered, ranging from ionic salts [15–16], over oxides [17–19], nitrides [20], and molecular layers [21–22] to 2D materials, such as graphene [23–24], and hexagonal boron nitride [25].

The most recent development of decoupling layers made use of the in situ fabrication of single layers of transition metal dichalcogenides on metal surfaces. A monolayer of MoS_2_ on Au(111) provided very narrow molecular resonances, close to the thermal resolution limit at 4.6 K [26]. The exquisite decoupling efficiency has been ascribed to a combination of its rather large thickness of three atomic layers, its electronic bandgap, and its non-ionic nature. Together, these properties prohibited fast electronic relaxations into the metal and coupling to phonons, which otherwise led to lifetime broadening [27–28].

The electronic properties of MoS_2_ on a metal surface are not the same as those of a free-standing monolayer. Both theory and experiment have found considerable hybridization of electronic states at the interface [29]. As a consequence, the bandgap is narrowed. Instead of the predicted bandgap of 2.8 eV of the free-standing layer [30–31], the bandgap of the hybrid structure amounts to only approx. 1.7 eV [29]. Interestingly, the states at the *K* point are much less affected than the states at the Γ point. Hence, the system remains promising for optoelectronic devices with selective access to the spin–orbit-split bands at *K* and *K*′ by circularly polarized light [32].

The potential as decoupling layer for molecules may become even more appealing by the fact that monolayers of transition metal dichalcogenides can be grown in situ on different metal surfaces, where the precise hybridization and band alignment depend on the nature of the substrate [33]. One may thus envision tuning the bandgap alignment for decoupling either the lowest unoccupied (LUMO) or the highest occupied molecular orbital (HOMO) of the molecules.

While MoS_2_ on Au(111) has already been established as an outstanding decoupling layer [26], we will now explore this potential for MoS_2_ on a Ag(111) surface. In agreement with the band modifications of WS_2_ on Au(111) and Ag(111), we find that the bandgap remains almost the same, albeit shifted to lower energies [33]. As a test molecule we chose tetracyanoquinodimethane (TCNQ). Due to its electron-accepting character, this choice will allow us to detect a negative ion resonance within the bandgap of MoS_2_. We will show that the LUMO is indeed decoupled from the metallic substrate as we can detect a narrow line width followed by a satellite structure. We can reproduce this fine structure by simulating the vibronic states of the gas-phase molecule.

## Results and Discussion

We have grown monolayer islands of MoS_2_ on an atomically clean Ag(111) surface, which had been exposed to sputtering–annealing cycles under ultrahigh vacuum before. The growth procedure was adapted from that of MoS_2_ on Au(111) [34–35], with Mo deposition on the surface in a H_2_S atmosphere of 5·10^−5^ mbar, while the sample is annealed to 800 K. TCNQ molecules were deposited on the as-prepared sample held at 230 K. The sample was then cooled down and transferred to the scanning tunneling microscope (STM). All measurements were performed at 4.6 K. Differential conductance (d*I*/d*V*) maps and spectra were recorded with a lock-in amplifier at modulation frequencies of 812–921 Hz, with the amplitudes given in the figure captions.

### Characterization of single-layer MoS_2_ on Ag(111)

**Figure 1:**
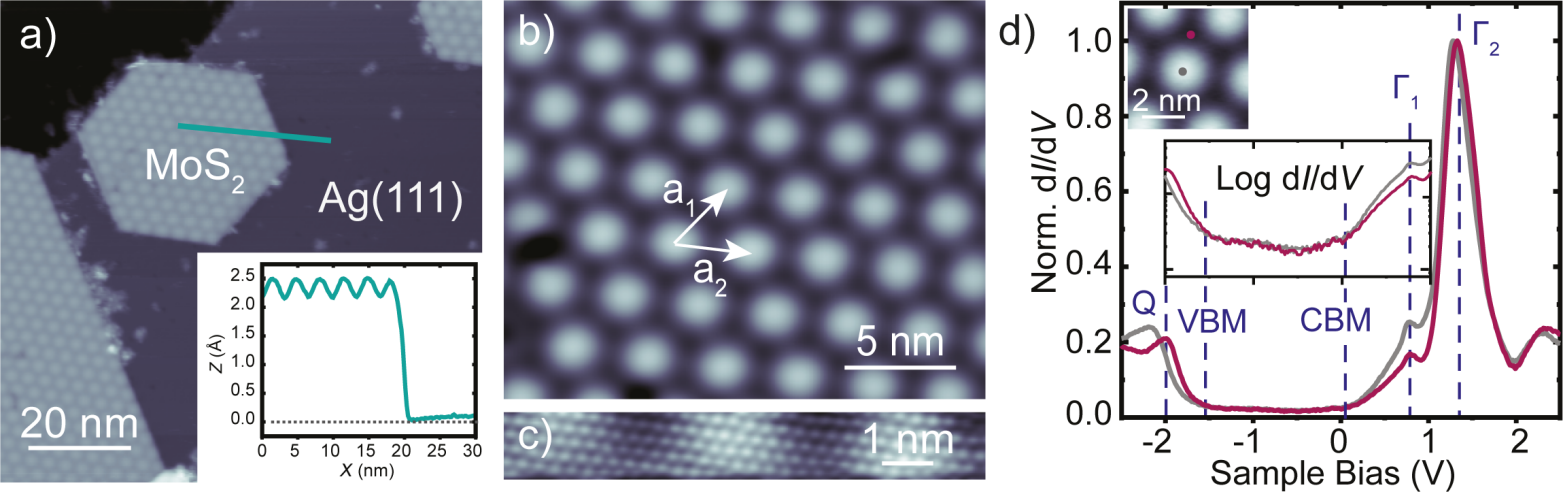
a) STM topography of MoS_2_ on Ag(111) recorded at *V* = 1.2 V, *I* = 20 pA. Inset: Line profile of a monolayer MoS_2_ island along the green line. b) Close-up view on the moiré structure. c) Atomically resolved terminating S layer (*V* = 5 mV, *I* = 1 nA). d) Constant-height d*I*/d*V* spectra of MoS_2_/Ag(111) recorded on a top and on a hollow region of the moiré structure as shown on the inserted STM topography (feedback opened at *V* = 2.5 V, *I* = 0.5 nA, *V*_mod_ = 10 mV). The inset shows the gap region of MoS_2_/Ag(111) on a logarithmic scale. We identify the valence band maximum (VBM) and the conduction band minimum (CBM) as the changes of the slope of the d*I*/d*V* signal. Dashed lines indicate the CBM at approx. 0.05 V and the VBM at approx. −1.55 V. The strong features in the d*I*/d*V* spectra are associated to the onset of specific bands, which are labeled by *Q*, Γ_1_ and Γ_2_ according to their location in the Brillouin zone. The assignment follows that in [38].

Figure 1a presents an STM image of the Ag(111) surface after the growth of MoS_2_ as described above. We observe islands with tens to hundreds of nanometer diameter and of 2.3 ± 0.2 Å apparent height (inset of Figure 1). The apparent height is much smaller than the layer distance in bulk MoS_2_ [36] due to electronic structure effects, but in agreement with a single layer of MoS_2_ on a metal surface [34]. The islands exhibit a characteristic hexagonal pattern reflecting a moiré structure, which results from the lattice mismatch between the Ag(111) surface and MoS_2_ (Figure 1b). Areas with large apparent height correspond to domains in which the S atoms sit on top of Ag atoms, whereas the lower areas represent two different hollow sites (fcc or hcp stacking) of the S atoms on the Ag lattice. The most abundant moiré periodicity amounts to approx. 3.3 ± 0.1 nm. This value is similar to the one observed for MoS_2_ on Au(111) [29,32,34,37].

Given the similar lattice constants of Au (4.08 Å) and Ag (4.09 Å), a locking into a similar superstructure at the metal–MoS_2_ interface is not surprising. However, occasionally, we also observe moiré patterns with lattice constants of 3.6 ± 0.1 and 3.0 ± 0.1 nm, and different angles between MoS_2_ and the Ag(111) lattice. This indicates shallow energetic minima of the lattice orientations. Atomically resolved STM images (Figure 1c) reveal the expected S–S distance of 3.15 Å in the top layer [36,39–41].

**Figure 2:**
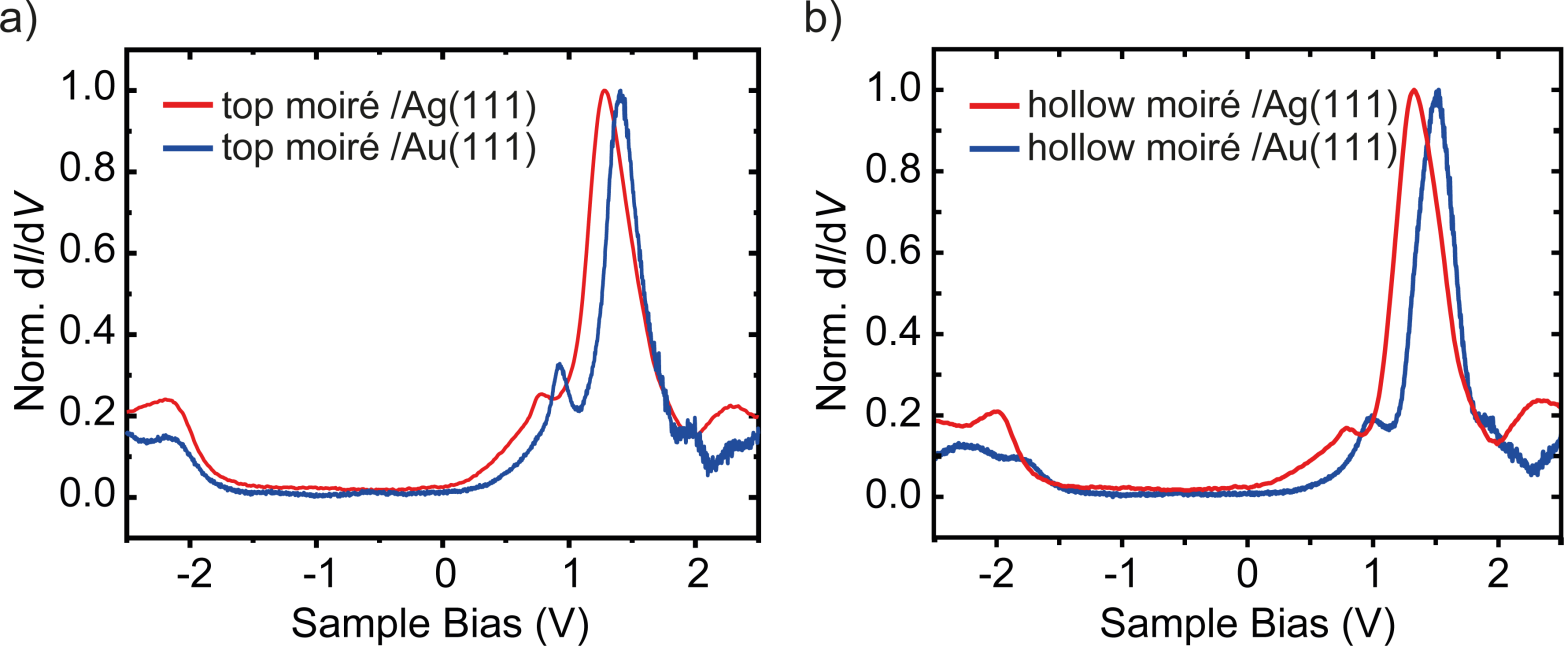
Constant-height d*I*/d*V* spectra recorded (a) on a top and (b) on a hollow site of the moiré structure of MoS_2_ on Ag(111) (red curves) and on Au(111) (blue curves). Feedback opened at *V* = 2.5 V, *I* = 0.5 nA, *V*_mod_ = 10 mV (all spectra, except for hollow site on Au(111): *V*_mod_ = 5 mV).

For an efficient decoupling of a molecule from the substrate, the interlayer must provide an electronic bandgap. As the moiré pattern bears a topographic and an electronic modulation [38], we investigate the differential conductance (d*I*/d*V*) spectra on different locations (Figure 1d). We first examine the spectrum on the top site of the moiré structure. We observe a gap in the density of states, which is flanked by an onset of conductance at approx. −1.55 V and approx. +0.05 V (marked by dashed lines labeled VBM or CBM, which have been determined from a logarithmically scaled plot). Additionally, there are pronounced peaks at approx. 0.77 V and approx. 1.28 V. First, we note that the observed bandgap is significantly smaller than the 2.8 eV bandgap of a single layer of free-standing MoS_2_ [30–31]. This indicates a strong hybridization of the electronic states of the MoS_2_ layer and the Ag substrate. Second, we note that the spectral features are similar to those observed for single-layer MoS_2_ on Au(111) [29,35,38]. For direct comparison, we plot the spectra on the top sites of the MoS_2_ moiré on Au(111) and Ag(111) in Figure 2a. At negative bias voltage, the onsets of conductance are essentially the same, while the features at positive bias voltage appear approx. 140 mV closer to the Fermi level on Ag(111) than on Au(111).

Before discussing the differences between the layers on Au(111) and Ag(111), we investigate the effect of the different stacking at the interface on the electronic properties. The spectrum of a hollow site on Ag(111) shows a shift of the features at negative bias voltage by about approx. 130 mV towards the Fermi level (*E*_F_), whereas the peaks at positive bias undergo a much smaller shift (approx. 50 mV) away from *E*_F_ (Figure 1d). On Au(111), there are also variations between hollow and top sites, with the strongest shift at negative bias voltage (Figure 2).

To understand the differences between the substrate and the local sites, we first discuss the origin of the spectroscopic features. Based on the similarity of the spectral shapes on Au(111) and Ag(111), we tentatively assign the strong peaks at approx. 0.8 V (labeled as Γ_1_) and approx. 1.3 V (labeled as Γ_2_) (values averaged over the different moiré sites) to bands at the 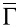 point [38]. More precisely, the peak at Γ_2_ has been assigned to bands at Γ, which are also present in free-standing MoS_2_, but are broadened due to hybridization with the substrate. The peak at Γ_1_ has been observed in tunneling spectra of MoS_2_ on Au(111), but has not been found in calculations. It has been interpreted as a hybrid metal–MoS_2_ or an interface state [38]. The conduction band minimum, which is expected to lie at the 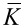 point for quasi free-standing as well as metal-supported single-layer MoS_2_ [29,42–44], is hardly visible in the tunneling spectra due to the rapid decay of the tunneling constant with *k*_∥_[38,45]. The same applies to the valence band maximum, such that the strongest feature in the tunneling spectra at −2 V arises from bands close to the 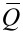 point [38].

Comparison of spectra on the moiré hollow sites suggest a rigid shift of the conduction bands between the MoS_2_ bands on Ag and Au. In a very simple interpretation, this agrees with the lower work function of Ag than that of Au. A down-shift of the conduction band structure by approx. 280 meV has been observed by photoemission of WS_2_ on Au(111) and Ag(111) [33]. Angle-resolved measurements further showed that the shift also included band distortions, such that bands at *Q* were crossing *E*_F_ (instead of at *K*). The band distortion was explained by hybridization of the WS_2_ bands with the Ag substrate [33]. As our d*I*/d*V* signal is not sensitive to *k*_∥_, we would not be able to detect band distortions in the MoS_2_–Ag system. However, the clear shift of the states at Γ can be easily understood by hybridization of S-derived states of mainly out-of-plane character with Ag states in analogy to [29].

In the occupied states, the bands on the hollow site follow the same trend of a down-shift, suggesting that the states near 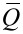 are equally affected by hybridization with Ag states [33]. In contrast, the tunneling spectra on the top sites, seem to coincide for Au and Ag substrates. We also note that the tunneling conductance close to the 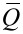 point is the most sensitive to the precise location on the moiré pattern. Hence, we suggest that this site is most strongly affected by screening effects, which may vary on the different substrates [46] and partially compensate for hybridization effects.

### Electronic properties of TCNQ molecules on MoS_2_ on Ag(111)

**Figure 3:**
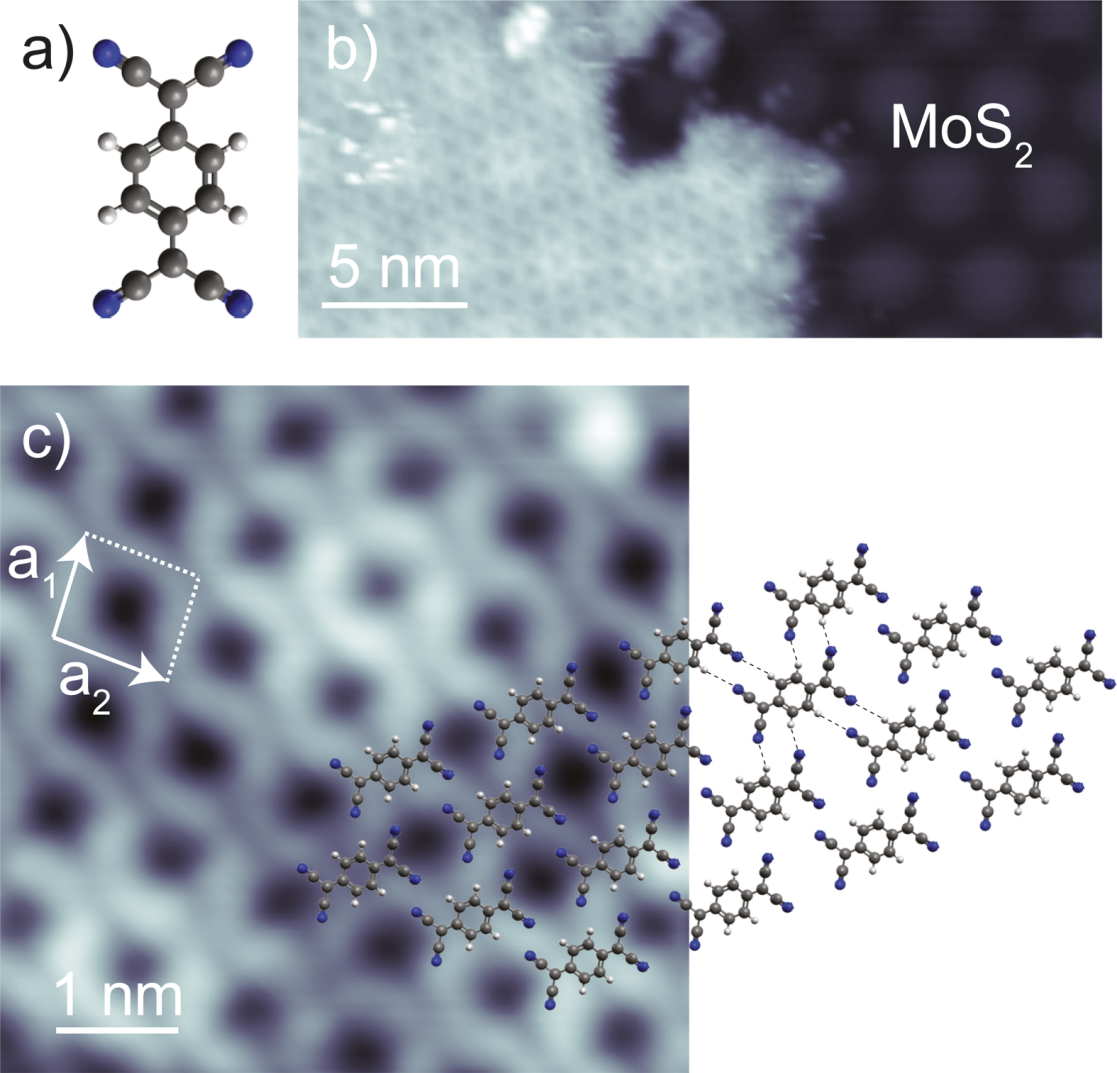
a) Stick-and-ball model of TCNQ. Gray, blue, and white spheres represent C, N, and H atoms, respectively. b) STM topography of a TCNQ molecular island on MoS_2_/Ag(111) recorded at *V* = 1 V, *I* = 10 pA. c) STM topography of a TCNQ island on MoS_2_/Ag(111) recorded at *V* = 0.8 V, *I* = 200 pA, with superimposed molecular models suggesting intermolecular dipole–dipole interactions (dashed lines). White arrows represent the unit cell of the self-organized TCNQ domain with lattice vectors *a*_1_ = 0.9 ± 0.10 nm and *a*_2_ = 1.0 ± 0.10 nm and the angle between them of (96 ± 2)°.

**Figure 4:**
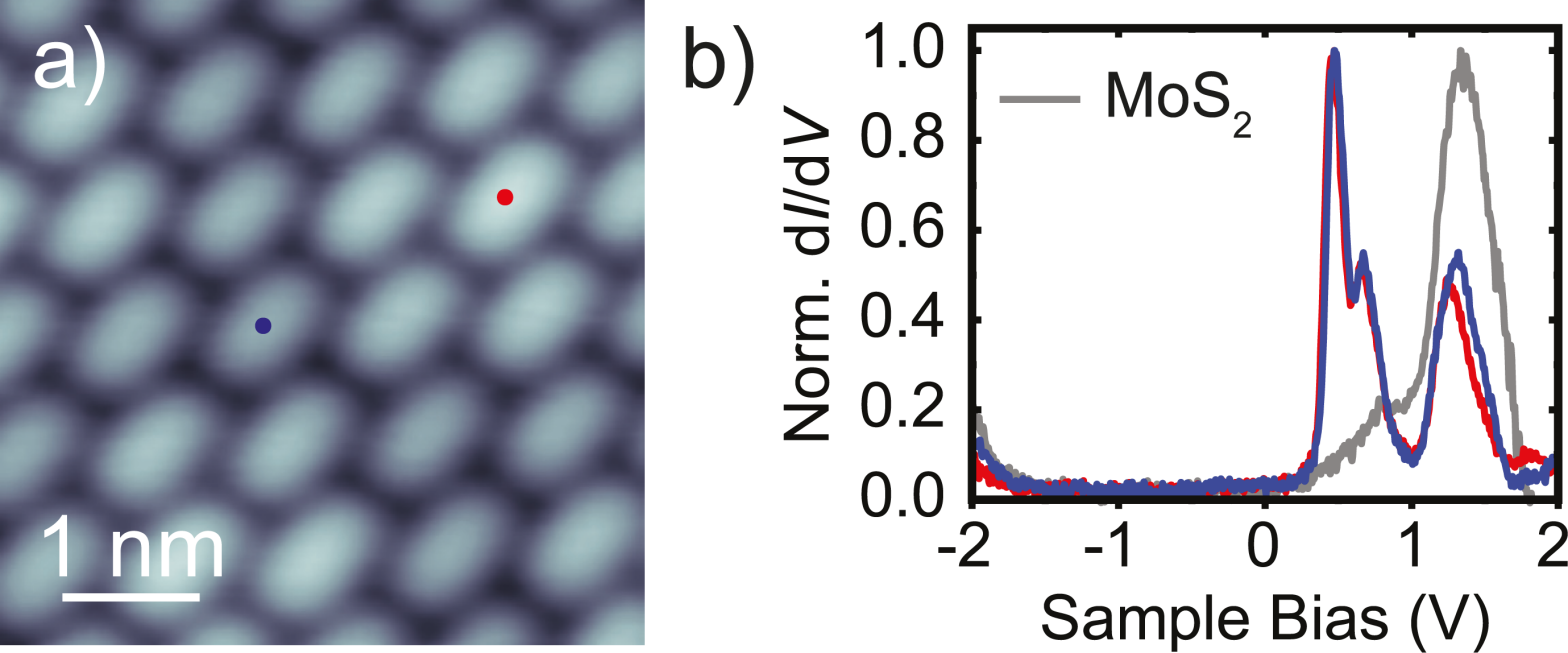
a) STM topography of a self-assembled TCNQ island on MoS_2_/Ag(111), recorded at *V* = 0.2 V, *I* = 20 pA. b) d*I*/d*V* spectra acquired on TCNQ molecules within the island in panel a, with the precise location marked by colored dots. The gray spectrum was recorded on a bare MoS_2_ layer for reference. Feedback opened at *V* = 2 V, *I* = 100 pA, with *V*_mod_ =20 mV.

Deposition of TCNQ molecules (structure shown in Figure 3a) on the sample held at 230 K leads to large densely packed molecular islands on the MoS_2_ areas (Figure 3b). The large size and high degree of order of these islands reflects a low diffusion barrier on the MoS_2_ substrate. The moiré pattern of MoS_2_ remains intact and visible through the molecular monolayer. High-resolution STM images recorded at 0.8 V (Figure 3c) allow for the resolution of the individual molecules and their arrangement. Each TCNQ molecule appears with back-to-back double U-shapes separated by a nodal plane. As will be discussed later, and based on previous work on TCNQ [5,23], this appearance can be associated to the spatial distribution of the lowest unoccupied molecular orbital (LUMO). The molecular arrangement can be described by the lattice vectors *a*_1_ = 0.9 ± 0.1 nm, *a*_2_ = 1.0 ± 0.1 nm and the angle (96 ± 2)° (see model in Figure 3c). This structure is stabilized by dipole–dipole interactions between the cyano endgroups and the quinone center of neighboring molecules. This assembly is very similar to typical self-assembled TCNQ islands on weakly interacting substrates [5,23,47–49]. When measured at a lower bias voltage (e.g., at *V* = 0.2 V in Figure 4a), the molecules appear with featureless elliptical shape, reflecting only the topographic extent of the molecules.

**Figure 5:**
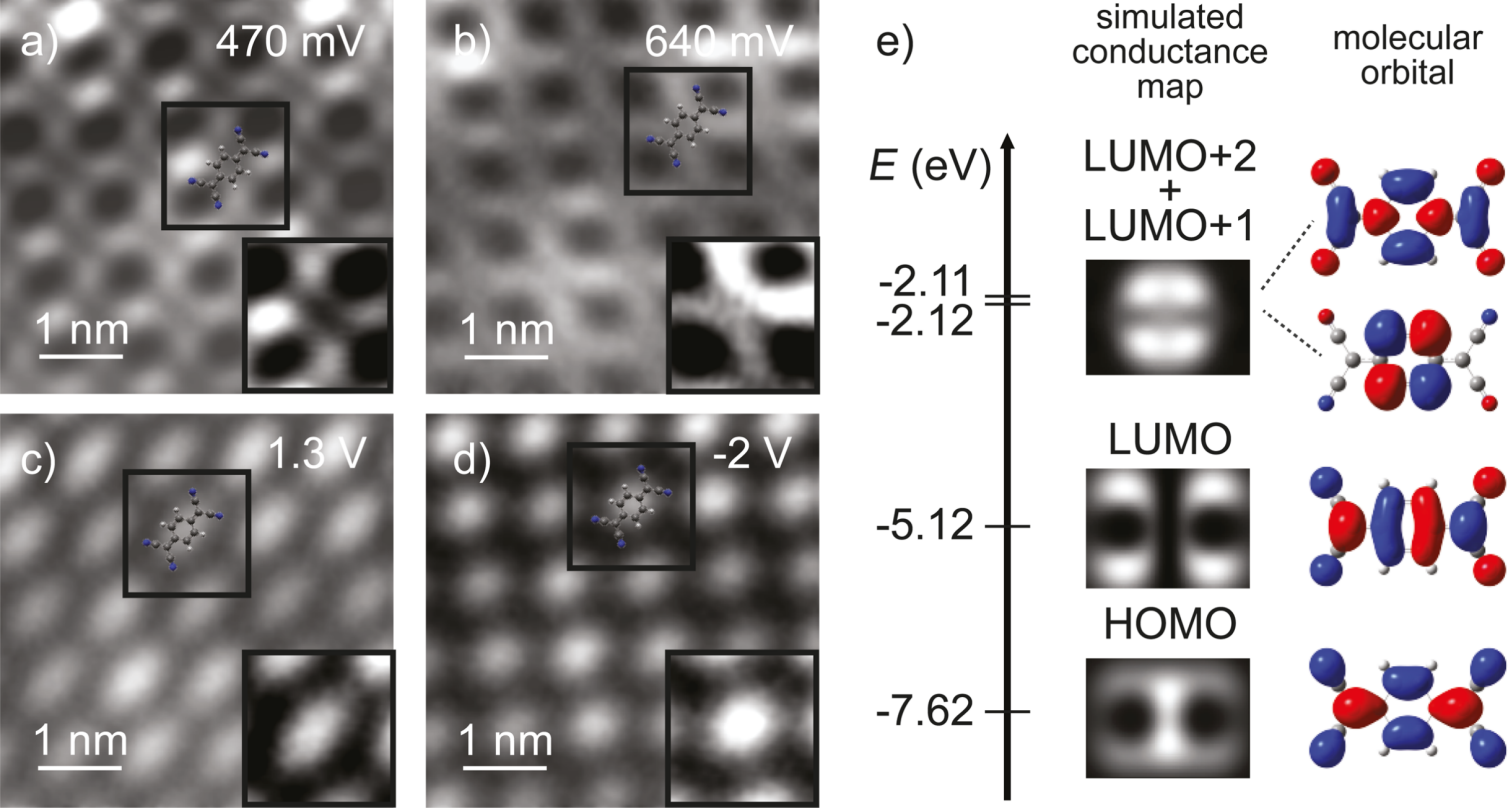
a–d) Constant-height d*I*/d*V* maps of a TCNQ island on MoS_2_ recorded at the resonance energies derived in Figure 4b. Feedback opened in panels (a–c) *V* = 2 V, *I* = 100 pA and (d) *V* = −2 V, *I* = 30 pA on the center of the molecule with *V*_mod_ = 20 mV. Close-up images with enhanced contrast on one molecule are shown as inset for each map. e) Energy-level diagram of TCNQ determined from gas-phase DFT calculations (left). The isosurfaces of the frontier molecular orbitals are shown on the right. These have been used to calculate the tunneling matrix element *M*_ts_ with an s-wave tip at a tip–molecule distance of 7.5 Å, work function of 5 eV. The map of the spatial distribution of 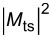 is shown in the middle panel.

The strong bias-voltage dependence of the TCNQ molecules on the MoS_2_ layer promises energetically well separated molecular states. To investigate these properties in more detail, we recorded d*I*/d*V* spectra on top of the molecules (Figure 4b). These show two main resonances at approx. 0.47 V and approx. 0.64 V. Another peak at approx. 1.3 V matches the Γ resonance of the bare MoS_2_ layer. At negative bias voltage, we observe an onset of conductance at approx. −1.8 V. The d*I*/d*V* spectra thus show that the STM image in Figure 4a was recorded within the energy gap of the molecule, which explains the featureless shape. In order to determine the origin of each of the resonances, we recorded constant-height d*I*/d*V* maps at their corresponding energies (Figure 5).

For the first resonance at positive bias voltage (470 mV, Figure 5a), we observe the same double U-shape, separated by a nodal plane, which we used in Figure 3 for the identification of the molecular arrangement. The d*I*/d*V* map at 640 mV exhibits the same shape, suggesting the same orbital as its origin. At 1.3 V, the molecules do not show any characteristic feature (Figure 5c). Finally, Figure 5d presents a conductance map at −2 V associated with the onset of conductance observed at negative bias voltage for spectra on the molecule. Here, the d*I*/d*V* signal is rather blurred, but we remark that it is more localized in the center of the molecule as compared to the elliptical shape in Figure 5c.

For the identification of molecular orbitals, it is often sufficient to compare the d*I*/d*V* maps with the shape of the gas-phase molecular orbitals. Using this method, the U-shaped features have previously been associated to the LUMO of TCNQ [5,23,49]. Here, we corroborate this assignment by simulating constant-height d*I*/d*V* maps of a free, flat-lying molecule. We first calculated the gas-phase electronic structure using density functional theory (DFT) calculations with the B3PW91 functional and the 6-31g(d,p) basis set as implemented in the Gaussian 09 package [50]. The isodensity contour plots of the highest occupied molecular orbital (HOMO) and some of the lowest unoccupied orbitals are shown in Figure 5e, right panel. The HOMO/LUMO can be unambiguously distinguished by the absence/presence of a nodal plane at the center of the quinone backbone. For direct comparison with the d*I*/d*V* maps, we calculate the tunneling matrix element between an s-wave tip and the spatially resolved molecular wave function across the molecule [51]. The maps of the square of the tunneling matrix element are depicted in Figure 5e next to the corresponding molecular orbitals. Because LUMO+1 and LUMO+2 are quasi-degenerate, we used the sum of their wave functions for the calculations of the tunneling matrix elements. As expected, the nodal planes of the molecular orbitals dominate the simulated d*I*/d*V* maps and can be taken as a robust signature for molecular orbital identification. Additionally, the simulated maps reveal that the d*I*/d*V* intensity is not directly proportional to the isosurface density. For instance, there is hardly any intensity within the U shapes of the TCNQ LUMO, and the HOMO is mainly localized at the very center of the quinone moiety. We note that the simulated maps were obtained at a tip–molecule distance (center of the s-wave tip to center of the molecule) of 7.5 Å. This value was chosen because it represents reasonable tunneling conditions in experiments. However, variation of the tip height by (±2 Å) does not have any influence on the observation of the main features within the map (i.e., nodal planes, or intensity maxima) [52].

Comparison with the experimental constant-height d*I*/d*V* maps, now allows for an unambiguous identification of the origin of the molecular resonances. As suggested previously, the resonance at 0.47 V can be derived from the LUMO with the double U-shape being in very good agreement with the calculations of the tunneling matrix element. The very same signatures in the conductance map at 0.64 V suggest that this resonance stems from the LUMO as well. The DFT calculations show that the LUMO is non-degenerate. Hence, we can exclude a substrate-induced lifting of the degeneracy. The energy difference of only 170 meV between the two resonances lies within the typical vibrational energies of organic molecules and may, thus, be indicative of a vibronic peak. We will elucidate this point further below.

The d*I*/d*V* map at 1.3 V essentially shows the same elliptical shapes of the molecules as the STM image recorded in the electronic gap (Figure 4a). Our DFT calculations suggest that the next higher unoccupied orbitals lie 3 eV above the LUMO and show a pattern of nodal planes that are absent in the experiment. Additionally, given the energy similar to that of the MoS_2_ bands, this resonance is probably not associated to the molecular layer, but to direct tunneling into the MoS_2_ states.

The assignment of the orbital origin at negative bias voltage bears some intricacies, because the experimental map lacks characteristic nodal planes. The reduced spatial resolution is most probably caused by the overlap with density of states of the substrate as we are approaching the onset of the valence band of MoS_2_. One may suggest that the stronger localization of d*I*/d*V* intensity toward the quinone center is in agreement with the large tunneling matrix element of the HOMO at the center of the molecule. This assignment may be enforced by the coincidence of the observed molecular energy gap of TCNQ with the DFT-derived gap. However, DFT is known to underestimate HOMO–LUMO gaps. Although this effect may be compensated by the screening properties of the substrate, we refrain from a definite assignment. In any case, our data clearly shows that the HOMO is at or within the conduction band of MoS_2_.

By comparison with simulations, we thus arrive at a clear identification of the energy level alignment. Most notably, we find that the LUMO-derived resonance lies close to, but above, the Fermi level of the substrate, whereas the HOMO is far below. This leaves the molecule in a neutral state with a negligible amount of charge transfer, despite the electron accepting character of TCNQ. Nonetheless, its electron affinity of approx. 3.4 eV [53–54] is consistent with the LUMO alignment just above *E*_F_ when considering the work function of MoS_2_/Ag(111) of 4.7 eV [55]. We found small shifts of the LUMO onsets by at most 50 mV between the spectra of TCNQ molecules lying at the top or hollow sites of the moiré structure of MoS_2_. These shifts correspond to the moiré-induced shifts in unoccupied states of the MoS_2_ layer and thus only reflect the different screening properties from the substrate. In turn, we do not observe any modification of the electronic structure of MoS_2_. This indicates weak interactions of the molecules all along the MoS_2_ layer.

Importantly, the resonance at 470 mV has a rather narrow width of approx. 100 mV. This is significantly smaller than resonances typically observed on metal surfaces, where strong hybridization effects lead to widths of the order of approx. 500 meV [5,48]. The narrow width thus reflects that MoS_2_ acts as a decoupling layer from the metal substrate. However, this resonance width is broader than what has been observed for the HOMO resonance of other organic molecules on MoS_2_ on Au(111) [26,52,56]. In contrast to those cases, where the HOMO lay well inside the electronic gap of MoS_2_, the LUMO of TCNQ is located right at the onset of the conduction band. This provides relaxation pathways for electrons tunneling into the LUMO, though still significantly less than on the bare metal.

### Vibronic excitations of TCNQ on MoS_2_ on Ag(111)

**Figure 6:**
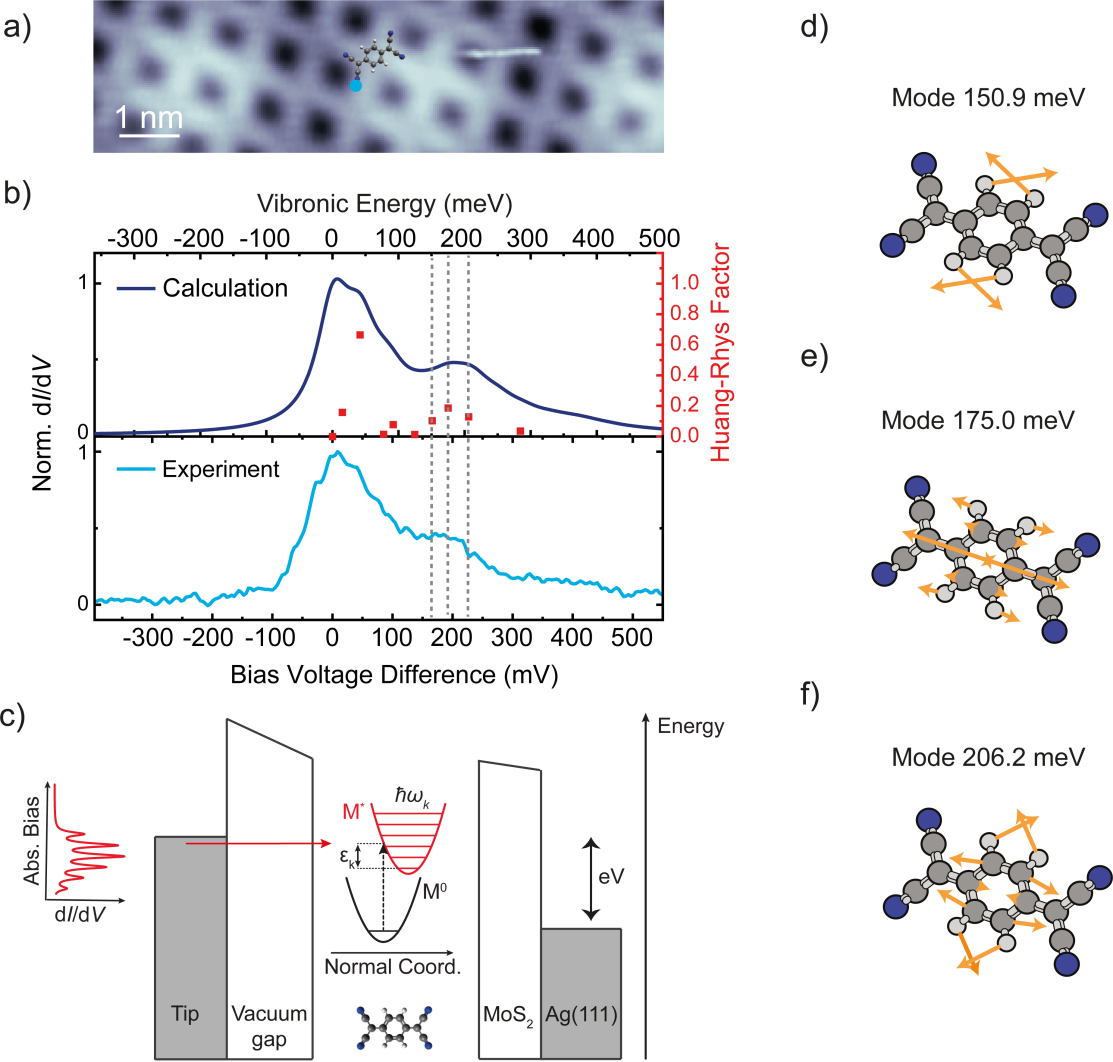
a) STM topography image of a TCNQ island recorded at *V* = 1 V, *I* = 10 pA. b) Simulated (top panel) and experimental (bottom panel) d*I*/d*V* spectra at the position indicated by the blue dot in panel (a) with feedback opened at *V* = 2 V, *I* = 100 pA, with *V*_mod_ = 10 mV. The simulated spectrum is obtained from DFT calculations for all vibrational modes of the TCNQ^−^ molecule with a Huang–Rhys factor higher than 0.01 (dots associated with the right axis). A Lorentzian peak of 60 meV broadening is applied to all of these modes. c) Schematic representation of electron transport through a TCNQ molecule adsorbed on MoS_2_/Ag(111): singly charged TCNQ^−^ is formed upon injecting an electron into a vibronic state of an unoccupied molecular electronic level. d–f) Visualization of the vibrational modes contributing to the satellite peak. The orange arrows represent the displacement of the atoms involved in these vibrations.

Having shown that the resonances at 470 and 640 mV originate both from the LUMO of TCNQ, we now turn to a more detailed analysis. A close-up view of the spectral range with these peaks is shown in the bottom panel of Figure 6b with the LUMO-derived peak at 470 mV shifted to zero energy and its peak height being normalized. The satellite structure is reminiscent of vibronic sidebands, which occur due to the simultaneous excitation of a vibrational mode upon charging [22,25,57–61]. The sidepeaks should thus obey the same symmetry as the parent orbital state [62–64]. In the simplest case, these excitations can be described within the Franck–Condon model (see sketch in Figure 6c). When probing the LUMO in tunneling spectroscopy, the molecule is transiently negatively charged. Within the Born–Oppenheimer approximation, this process is described by a vertical transition in the energy level diagram from the ground state *M*^0^ to the excited state M*. Upon charging, the molecule undergoes a geometric distortion, captured by the shift of the potential energy curve of the excited state. Vertical transitions allow for probing many vibronic states, with the intensities given by a Poisson distribution,


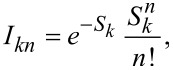


with *S_k_* being the Huang–Rhys factor of the vibrational mode *k* and *n* its harmonics. The Huang–Rhys factor is determined by the relaxation energy ε*_k_* of a vibrational mode when charging the molecule as


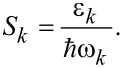


From the DFT calculations of the TCNQ molecule, we determine all vibrational eigenmodes in the negatively charged state and also derive the Huang–Rhys factors *S_k_* [26]. The latter are plotted in the upper panel of Figure 6b (dots, right axis). Applying to each of the vibronic states a Lorentzian peak with a full width at half maximum of 60 meV and intensity proportional to the Poisson distribution, as described above, leads to the simulated Franck–Condon spectrum in the upper panel of Figure 6b. This spectrum closely resembles the experimental one and, therefore, nicely reflects the nature of the satellite structure. We note that the bias voltage axis (bottom panel) is scaled by 10% compared to the energy axis (top panel) to account for the voltage drop across the MoS_2_ layer [65]. We now realize that the peak at approx. 640 meV consists of three vibrational modes (at 151, 175, and 206 meV) exhibiting a large Huang–Rhys factor. These modes correspond to in-plane breathing modes of TCNQ (see schemes in Figure 6d–f), which are particularly sensitive to charging. Additionally, a mode at 40 meV has a large Huang–Rhys factor. The excitation of this mode is not energetically well separated from the elastic onset of the LUMO in experiment. However, this mode contributes to an asymmetric line shape, which can be realized by comparing the low-energy flank to the high-energy fall-off of the first resonance. The low-energy side can be fitted by a Voigt profile and suggests a lifetime broadening of 55 ± 15 meV. This is, however, insufficient for a peak separation from the mode at 40 meV.

We further note that the experimental spectrum was taken on a cyano group, where no nodal planes exist in the LUMO, as their presence may lead to vibration-assisted tunneling in addition to the bare Franck–Condon excitation [52].

## Conclusion

We have shown that a single layer of MoS_2_ may act as a decoupling layer for molecules from the underlying metal surface, if the molecular resonances lie within the semiconducting bandgap of MoS_2_. MoS_2_ on Au(111) and Ag(111) exhibit very similar gap structures, but are shifted in energy according to the different work functions of the metal. Though this is not the only reason for the band modifications [33], we suggest that such considerations may help when searching for appropriate decoupling layers for specific molecules. We have challenged the decoupling properties of MoS_2_/Ag(111) for TCNQ molecules. These exhibit their LUMO resonance just at the conduction band onset of MoS_2_, whereas the HOMO lies within the valence band. Hence, the HOMO is not decoupled from the substrate, and also the LUMO suffers considerable lifetime broadening as compared to resonances, which would be well separated from the onsets of the MoS_2_ bands. The lifetime broadening of 55 ± 15 meV can be translated into a lifetime of approx. 6 fs of the excited state. This is almost one order of magnitude longer than on the bare metal surface, where the hot electron vanishes into the bulk on ultrafast timescales, but an order of magnitude shorter than for molecular resonances well separated from the band onsets [26,52,56]. Yet, the increase in the lifetime of the excited state allowed us to resolve vibronic states of the transiently negatively charged TCNQ molecule albeit only up to approx. 200 meV above the LUMO resonance, where contributions of MoS_2_ bands at Γ become strong. Our simulations reproduce the experimental satellite structure of the LUMO very well, although the experimental width prevented us from resolving the individual modes.
